# Estimation of Occupancy Using IoT Sensors and a Carbon Dioxide-Based Machine Learning Model with Ventilation System and Differential Pressure Data

**DOI:** 10.3390/s23020585

**Published:** 2023-01-04

**Authors:** Jehyun Kim, JongIl Bang, Anseop Choi, Hyeun Jun Moon, Minki Sung

**Affiliations:** 1 Department of Architectural Engineering, Sejong University, 209 Neungdong-Ro, Gwangjin-Gu, Seoul 05006, Republic of Korea; 2 Department of Architectural Engineering, Dankook University, Youngin 16890, Republic of Korea

**Keywords:** internet of things sensor, differential pressure, carbon dioxide, occupancy, ventilation system, machine learning

## Abstract

Infectious diseases such as the COVID-19 pandemic have necessitated preventive measures against the spread of indoor infections. There has been increasing interest in indoor air quality (IAQ) management. Air quality can be managed simply by alleviating the source of infection or pollution, but the person within a space can be the source of infection or pollution, thus necessitating an estimation of the exact number of people occupying the space. Generally, management plans for mitigating the spread of infections and maintaining the IAQ, such as ventilation, are based on the number of people occupying the space. In this study, carbon dioxide (CO_2_)-based machine learning was used to estimate the number of people occupying a space. For machine learning, the CO_2_ concentration, ventilation system operation status, and indoor–outdoor and indoor–corridor differential pressure data were used. In the random forest (RF) and artificial neural network (ANN) models, where the CO_2_ concentration and ventilation system operation modes were input, the accuracy was highest at 0.9102 and 0.9180, respectively. When the CO_2_ concentration and differential pressure data were included, the accuracy was lowest at 0.8916 and 0.8936, respectively. Future differential pressure data will be associated with the change in the CO_2_ concentration to increase the accuracy of occupancy estimation.

## 1. Introduction

As people spend a significant amount of time indoors, it is important to manage indoor air quality (IAQ). Numerous research studies on the management of airborne pollutants such as carbon dioxide (CO_2_), particulate matter (PM), and volatile organic compounds (VOCs) are in progress [1]. In addition, efforts to prevent the spread of indoor infectious diseases have been made since the COVID-19 pandemic and are ongoing [2]. IAQ management aims to identify and enforce an appropriate standard value for every country. Typical management measures include the installation of ventilation systems or the use of air purifiers for the active ventilation of indoor spaces. Recently, ultraviolet sterilization has been adopted to reduce indoor pollutant concentrations, facilitating a response based on various physical–chemical and biological measures. However, indiscriminate or excessive responses can cause energy wastage [3,4]. Buildings account for approximately 30% of the total energy consumption. Estimating the number of occupants is essential for providing a suitable indoor environment and reducing the energy consumption [5,6,7,8,9,10].

Energy wastage needs to be mitigated and various relevant environmental factors efficiently managed. To identify these environmental factors, the indoor environment can be evaluated using suitable measurement equipment. However, IAQ measurement equipment is expensive and its utilization has numerous restrictions, such as size. In this study, a relatively inexpensive and small internet of Things (IoT) sensor that measures indoor air quality and differential pressure was used. However, these sensors are a follow-up measure for circumstances in the indoor environment that should be predicted, mitigated, and managed. In addition, unspecified environmental factors such as leakage and the accessibility for people complicate the real-time management of air quality. Considering that this cannot be seen as a steady state in terms of engineering, a method of estimating the IAQ as a preliminary prediction through machine learning is required. Estimation through machine learning can facilitate IAQ management by identifying indoor pollutants and contribute to energy saving by predicting the number of occupants.

This study aims to predict the number of people by using the differential pressure data and data from the ventilation system operation modes based on CO_2_ concentration, through data acquisition and machine learning in a living-lab using IoT sensors. Differential pressure is an indicator of air flow between rooms and pollutants, and air movement between spaces. This study identified the effect of indoor–outdoor and indoor–corridor differential pressure data on the prediction of occupants using CO_2_-based machine learning and the ventilation system operation. In general, environmental factors such as temperature and humidity are used for occupancy estimation. In order to accurately estimate the number of occupants, the differential pressure between spaces considering the entry and exit of people was used to improve accuracy. In addition, in the previous research on occupancy estimation, the presence or absence of the ventilation system in most spaces is different, but it is not reflected in the machine learning for occupancy estimation. Therefore, in this study, it was confirmed that variables such as the differential pressure and the ventilation system operation can affect occupant estimation. Furthermore, the present study reflects the influence of the ventilation system operation; therefore, providing a reference for IAQ management and infection prevention measures according to the estimated occupancy. An appropriate and efficient IAQ management plan for the target space can be based on the predicted number of occupants.

## 2. Literature Review

Various research studies such as Table 1 have estimated the number of occupants based on CO_2_ concentration. Certain studies estimate the number of occupants by measuring noise, illumination, lighting energy load, and Wi-Fi connection information among other factors from the environmental data on CO_2_ concentration and the indoor temperature and humidity (Table 1).

Masood et al. [5] estimated the CO_2_ concentration, indoor temperature, humidity, and absolute pressure using the extreme learning machine (ELM) model. Chen et al. [6], input the CO_2_ concentration, indoor temperature, humidity, and absolute pressure data into various models such as ELM, support vector machine (SVM), artificial neural network (ANN), linear discriminant analysis (LDA), K-nearest neighbors (KNN), and classification and regression tree (CART), and compared the model accuracies. The two studies above categorized occupancy into low (0–5), medium (6–10), and high (11–15) density depending on the number of occupants. Brennan et al. [7] determined the CO_2_ concentration, temperature, and humidity based on 0 to 4 occupants using the gradient boosting (GB), KNN, LDA, and random forest (RF) models. Data were measured using temperature, humidity, and CO_2_ sensors based on a raspberry pie board. Owing to the lack of data, this study compared accuracy levels by categorizing occupancy into ranges before determining the exact number of occupants.

According to Jiang et al. [8], the CO_2_ concentration may spike momentarily depending on the measurement noise, the irregular indoor air flow, and the occupants who irregularly approached the sensor; therefore, the CO_2_ concentration data was smoothly transformed and used. Ryu et al. [9] estimated the number of occupants by determining the CO_2_ concentration and average CO_2_ concentration for 15 min, the average change in CO_2_ concentration within 15 min, and the ratio of indoor to outdoor CO_2_ concentration using CART. As the CO_2_ concentration continuously changed owing to the indoor air flow and location of the occupant and the sensor, among other factors, the value obtained by reducing the instantaneous change was input based on the concentration average within 15 min. Zuraimi et al. [10] reduced the instantaneous spike effect of CO_2_ using a 5-min average concentration. Zhou et al. [11] estimated the concentration of CO_2_ using the GCForest, SVM, CART, and iHMM models. Noise was removed from the raw graph of the CO_2_ concentration through analysis. Before this study was conducted, the amount of change in the CO_2_ concentration and the CO_2_ concentration itself were initially designated as input variables. At that point, the number of occupants had not been accurately determined, and owing to the noise of the change in the concentration of CO_2_, there was one case where there was no occupant despite a high concentration of CO_2_.

Candanedo et al. [12] estimated the number of occupants using the LDA, CART, RF, and gradient boosted model (GBM) for the CO_2_ concentration, illumination, temperature, and humidity. The accuracy was compared using various data combinations based on the different models. Singh et al. [13] estimated the occupancy using data from the CO_2_ concentration, temperature, lighting energy consumption, passive infrared (PIR), and noise sensors and based on the LDA, QDA, SVM, and RF models. Adeogun et al. [14] estimated the CO_2_ concentration, temperature, humidity, pressure, total VOCs, and the sound and window state using the FNN model. The data collected in two rooms were trained using different models, and the accuracy was cross-validated by applying the learned models to different rooms. Elkhoukhi et al. [15] estimated the number of occupants using the LDA, RF, and CART models to analyze the CO_2_ concentration, lighting energy consumption, temperature, and humidity. The energy consumption was a suitable input variable for the occupancy estimation; however, preliminary preparation was needed to measure the energy consumption of the room. This study excluded energy consumption as a variable during the estimation of occupants in various rooms with mechanical ventilation, but aimed to later measure energy consumption to compare accuracy levels and integrate energy consumption as an input variable using smart plugs.

Wang et al. [16] estimated the number of occupants using the CO_2_ concentration, temperature, humidity, and Wi-Fi connection data. In a laboratory used by up to 25 people, the number of occupants was modeled at 5-min intervals during working hours (08:30–19:00). Hobson et al. [17] estimated the number of occupants using the ANN model for the CO_2_ concentration, Wi-Fi connection, PIR, and energy consumption analysis. To prevent the artificial inflation of the prediction accuracy of the model, the measured data obtained at the time of occupancy (between 06:00–22:00) was considered. This study estimated the number of occupants using various combinations of input variables. The accuracy of occupant estimation increased drastically when Wi-Fi connection information was added. Wang et al. [18] estimated the number of occupants using the ANN model to analyze environmental data and Wi-Fi access information. Accuracy according to the combination of the different sensor data was compared. Increasing the input factor did not always increase the accuracy. However, the Wi-Fi-access information was used with caution owing to privacy concerns. In addition, the exact location of the room could not be determined by connecting to the Wi-Fi around the living-lab, which presented a challenge. Upon solving this problem, our future study will consider the Wi-Fi information as one of the input variables and will compare the resulting effect of this inclusion on the accuracy of the estimations.

The occupancy estimation can be applied to various fields targeting buildings. The number of occupants acts as an important factor in a building performance simulation evaluation. Tekler et al. [28] proposed several techniques to accurately identify the dynamic movement patterns of occupants indoors. Among them, BEL (Bluetooth Low Energy, Bengaluru, India) technology using a mobile phone was used to confirm the location of occupants and a method for estimating the number of occupants using machine learning was presented. In addition, the occupant prediction can save energy consumption by reducing unnecessary HVAC operation in the space. Dong et al. [29] conducted a study on occupancy estimation techniques for the efficient operation of an HVAC in a building. Tekler et al. [30] proposed a method using an IoT-based smart plug for efficient energy management through the occupancy estimation, and facilitated energy reduction and occupancy satisfaction in the field for 5 months. Occupancy prediction data can be used in hospital facilities(emergency). Whitt et al. [31] developed an aggregation probability model of an emergency department (ED) through ongoing research. This model is used during the patient arrival process in the emergency room through a real-time prediction considering the patient’s arrival and departure time. Occupancy estimation data may be used in hospital facilities for different purposes. Littig et al. [32] conducted a study estimating the number of occupants in medical facilities. By estimating the number of occupants entering and exiting the hospital and the number of occupants at the hospital, various measures to cope with different concerns, such as the placement of medical staff, management of beds and the use of ambulances, were proposed.

**Figure 1:**
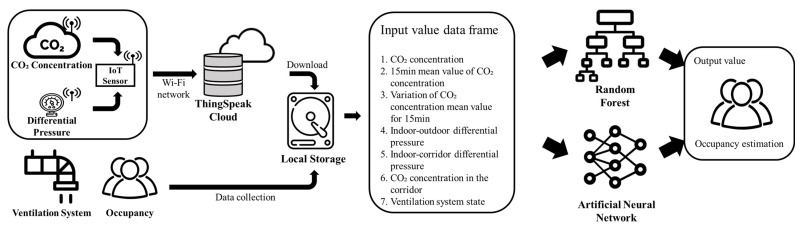
An overview of the process of the proposed study.

Most of the studies estimating the number of occupants based on the CO_2_ did not include a ventilation system in the chamber for data acquisition nor add related information as input variables, even when the ventilation system was activated. Considering that buildings use heating, ventilation, and air conditioning for mechanical ventilation, the change in the indoor CO_2_ concentration according to the operation of the ventilation system varies. In addition, in facilities such as negative-pressure isolation wards, the pressure difference between the rooms is set manually to control the airflow and prevent the infectious bacteria from leaking to the outside. Depending on the airtightness of the room and the pressure difference between the indoor and outdoor environments, air pollutants can be introduced by leakage. Based on this information, the change in the indoor CO_2_ concentration may vary according to the number of occupants, the ventilation system operation, and the differential pressure. Therefore, we intend to estimate the number of occupants based on the CO_2_ concentration through machine learning by reflecting various indoor environmental factors, the ventilation system operation, and the pressure difference between the room and the surrounding space. This is intended to facilitate the comparison and determination of possible contributions of the ventilation system operation, and the pressure difference between the room and surrounding space, to the accuracy of the occupant estimation based on machine learning. The entire process of the study is shown in Figure 1.

## 3. Materials and Methods

### 3.1. The Internet of Things Environmental Sensors

**Figure 2:**
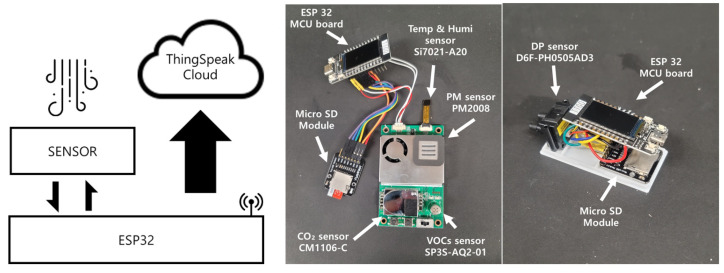
The concept of data mining and sensor configuration.

The integrated sensor modules of the IoT sensor were PM2.5, PM10, CO_2_, temperature, humidity, and VOCs. The data were set to be stored in ThingSpeak (MathWorks, Natick, MA, USA). The differential pressure sensor was D6F-PH0505AD3, and the environmental sensor AM1008W module. The AM1008W module used the CO_2_ sensor and PM sensor. The Si7021-A20 sensor was used for temperature and humidity. The SP3S-AQ2-0 was used for VOCs. A TTGO-T-Display ESP32 board was used as a micro control unit (MCU). In addition, the sensor was configured to store data as a sub whenever there was a communication error using the micro-SD module. Figure 2 shows the schematic of the sensor configuration. The sensor specifications are shown in Table 2. The accuracy of the differential pressure sensors was compared using a DG-700 (The Energy Conservatory-TEC, Minneapolis, MI, USA). The CO_2_ sensor calibration was performed at the same time from the outside for both the indoor and corridor sensors.

### 3.2. Data Mining and Analysis

**Figure 3:**
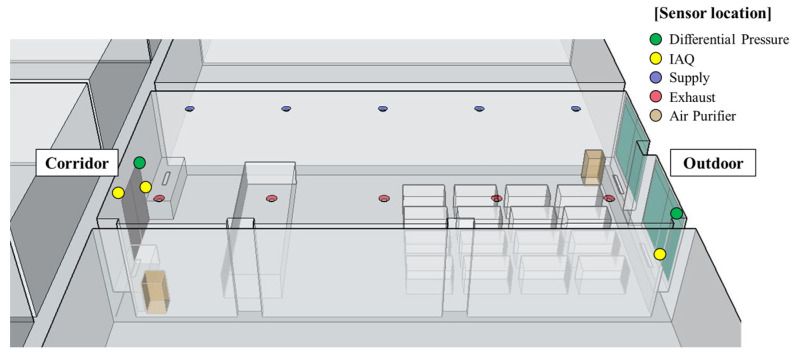
The sensor locations in the living-lab.

The volume of the living-lab shown in Figure 3 was 264 m^3^(14.8 × 6.6 × 2.7) and an air purifier AX90T7020WFD (SAMSUNG, Seoul, Korea) with an area capacity of 90 m^3^ was placed at the entrance and window. In addition, a heat recovery ventilation system was installed on the ceiling. The heat recovery HRD-EP250IBN (HIMPEL, Hwaseong-si, Korea) was applied and the air volume of this model was 230 CMH in a slight wind, 250 CMH in a moderate wind, and 270 CMH in a strong wind. The differential pressure and IAQ sensors were installed on the entance and window, respectively, to collect data. The maximum number of occupants using the living-lab was 17 during the measurement period. The machine learning model used a random forest (RF) and an artificial neural network (ANN). Data were acquired from 13 September to 22 November. Indoor environmental factors such as the CO_2_, the PM, the indoor–outdoor and indoor–corridor differential pressure, the number of occupants, the ventilation system, and the air purifier operating mode were measured. The data used for learning were collected for 55 days—13 September to 6 November—and data that was not collected due to Internet errors was excluded. Low, R et al [33] and Stekhoven et al [34] were used various methods of interpolating missing data. However, in this paper, among the data from 13 September to 6 November used for learning, from 11:52 on 16 September to 13:57 on 21 September, the input values of differential pressure and CO2 concentration data were not measured due to an internet connection error. It was possible to manually enter the ventilation system operation mode and the number of occupants, but the most important CO_2_ concentration, the average CO_2_ concentration, and the amount of change in the average CO_2_ concentration cannot be used. In addition, data that were collected from 11 pm to 8 am and over the weekends were disregarded because the living-lab was not in use at the time.

The CO_2_ concentration, the mean value of the CO_2_ concentration for 15 min (currently and at 15 min previously), the amount of change in the mean value of the CO_2_ concentration for 15 min, the indoor–outdoor and indoor–corridor differential pressure, and the ventilation system operation mode were set as the input values.

The measured CO_2_ concentration was significantly noisy; however, the noise could be reduced by using the mean value of the CO_2_ concentration for 15 min and the amount of change in the mean value of the CO_2_ concentration for 15 min. The ventilator operation mode was classified as 0 for being turned off, 1 for weak, 2 for moderate, and 3 for strong. In the operation mode of the ventilation system, the time when change occurred and the operation mode were hand-written and later entered into the model. For the differential pressure, data representing the 1-min mean value were used. For the occupancy data, the time and number of occupants were hand-written whenever the living-lab was used for classes, meetings, and seminars, for example. In addition, the accuracy was compared using the data from 7 November to 22 November, which were not used for learning. The data used for verification were disregarded because the accuracy would be excessively high if data from 11 p.m. to 8 a.m. on weekends were considered.

The cases were largely categorized according to whether the ventilation system was activated; consequently, the indoor–outdoor and indoor–corridor differential pressure, and the CO_2_ concentration of the corridor were used for machine learning. Subsequently, the accuracy was compared according to the operation mode data of the ventilation system and differential pressure. The case included: CO_2_ concentration, the mean value of the CO_2_ concentration within 15 min, and the amount of change in the mean value of the CO_2_ concentration within 15 min. The case is shown in Table 3. The input values were configured as shown in Table 3. The output value was the occupancy range and number of occupants.

The ventilation system operation mode was changed every Monday from 13 September to 6 November, when learning data were acquired because the ventilation system in the living-lab could not be controlled remotely. Moreover, data according to each ventilation operation mode were acquired. When acquiring data for verification, the ventilation system operation mode was arbitrarily set, and the data were obtained by manually entering the setting.

### 3.3. The Comparison of the Accuracy Factor and Machine Learning

The machine learning model was configured using the ANN in the Python 3.9.13 version Tensorflow library and the Scikit-learn’s RF library. The input values were set differently for each case, and the output values were set as occupancy range and number of occupants. The ANN model constructed in this study was structured as follows. The input layer set a node according to the number of input data, according to the case. In the case of the hidden layer, the number of nodes was set at 20. The occupancy ranges were set at 0 to 5, 6 to 10, 11 to 15, and 16 to 20. Of the total data used for machine learning (ANN and RF), 70% was used for learning and 30% for a self-test to verify the learning model. 

The ANN model using the Tensorflow library was composed of one input layer, two hidden layers, and one output layer. The Rectified Linear Unit (ReLU) function, the most commonly used activation function in most ANN models, was used as an activation function of the input and hidden layer. The ReLU function outputs 0 if the input value is less than 0, and outputs the input value as it is if the input value is greater than 0:

$$R\left(x\right)=\max\left(0,x\right)$$

The Softmax function that is most commonly used in the multi-classification model was used as the activation function of the output layer and the node was set to 1. The Softmax function normalizes the output value between 0 and 1. For this function, the sum of all output values is always 1:

$$\sigma {\left(Z\right)}_{i}=\frac{e^{Z_{i}}}{\sum_{k=1}^{K}e^{Z_{k}}}$$

Adam was used as an optimizer and the loss function was set to a categorical cross entropy. The metrics was set to accuracy.

In the case of the RF using the Scikit-learn’s library, a classifier was used. The maximum depth was set to 10 to prevent the overfitting or underfitting of the accuracy differences in the learning data, testing, and verification during the RF learning.

The actual and estimated number of occupants were compared using the Accuracy and the root mean square error (RMSE). The RMSE was calculated based on the difference between the actual values of N data and the estimated values of the model. The estimated value of occupants in the RMSE was obtained from the number of occupants, with a maximum interval value of 17:

$$\mathrm{RMSE}=\sqrt{\frac{\sum {\left(Y_{real}-Y_{predict}\right)}^{2}}{N}}$$

The Accuracy compares the actual and model-estimated number of occupants by dividing the intervals. The number of occupants is divided into four ranges: 0 to 5, 6 to 10, 11 to 15, and 16 to 20 to determine the range that includes the actual and estimated number of occupants. It is calculated using the range of the actual number of occupants by N data and the range of the estimated number of occupants:

$$\mathrm{Accuracy}\;=\frac{\sum True\;positive}{N}$$

## 4. Results

### 4.1. The Random Forest and Artificial Neural Network Train and Test Set

To derive an accurate output value from the decision tree model, data correlated with the output value should be analyzed and added as an input value. Therefore, factors that may affect the indoor CO_2_ concentration were identified to be the occupants, the ventilation system, and the inflow into the room from the surrounding space. The possible contribution of the differential pressure and CO_2_ concentration between the ventilation system operation mode and the surrounding space to the increased accuracy of occupancy prediction was examined. The Accuracy using the learning data from the RF and ANN as based on data from 13 September to 6 November, was compared for each case using the Accuracy and RMSE. The Accuracy divided the range according to the number of occupants and compared the range of the actual and estimated number of occupants. The RMSE used the actual and estimated numbers of occupants. Table 4 shows the results of the test using 30% of the data after learning, at 70% of the model data. However, when comparing the test accuracy by distinguishing 70% and 30% of the learning data, the accuracy can be excessively heightened if 30% of the test data includes a lot of sit-in-free situations. This necessitates the verification of accuracy using additional data not used for learning and testing.

### 4.2. The Random Forest and Artificial Neural Network Verification Set

**Figure 4:**
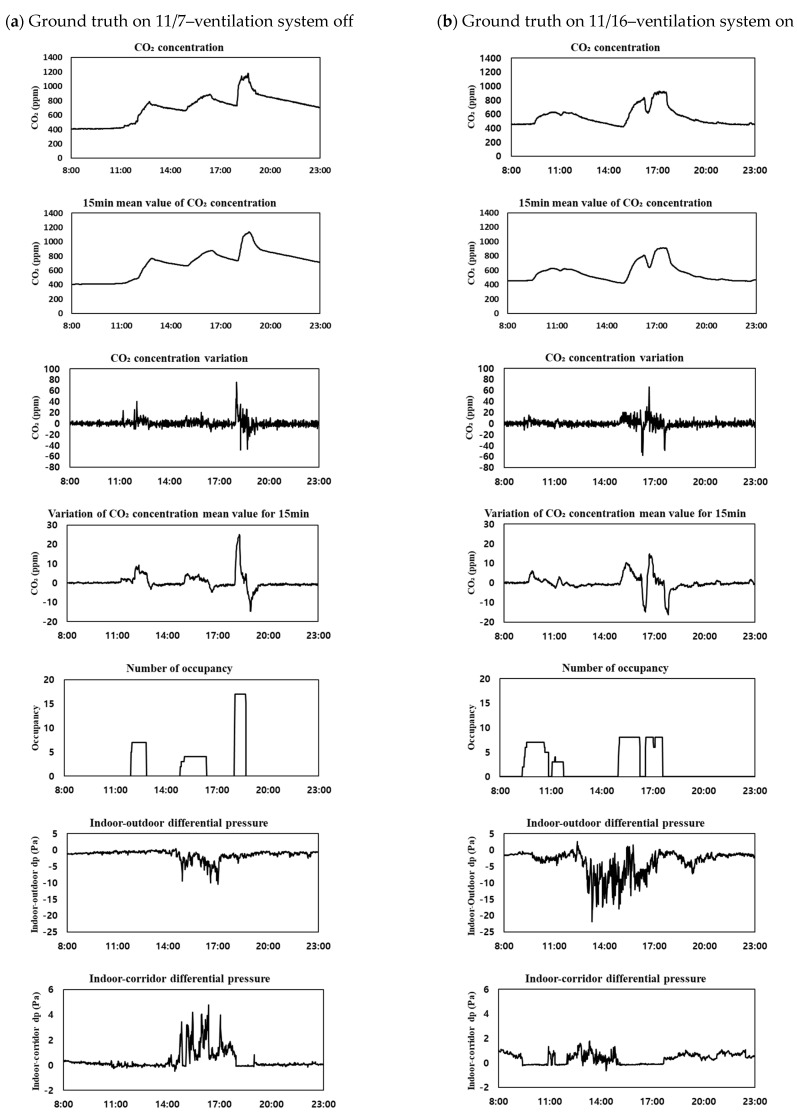
The ground truth data on (**a**) 11/7 and (**b**) 11/16.

To validate the trained model, the data set was divided into two parts. The Accuracy and RMSE for each case were compared in each data set. Data were measured when the ventilator was not operated from 7 November to 14 November. From 15 November to 22 November, the ventilation device was used arbitrarily. Depending on the date and time, whether the ventilation system was turned off or whether it was operated with a slight wind or a strong wind was measured. As the first data set, the data from 7 November to 14 November were used as one verification data set. As the second data set, the period from 7 November to 22 November was used as a validation data set. Among the data used for the accuracy verification, the CO₂ concentration, the mean value of the CO_2_ concentration within 15 min, the amount of change in the mean value of the CO_2_ concentration within 15 min, the number of occupants, the indoor–outdoor differential pressure, and the indoor–corridor differential pressure on 7 November, when the ventilation system was not operated, are shown in Figure 4a. The data on the day when the ventilation system was randomly operated, on 16 November, are shown in Figure 4b. The CO_2_ concentration graphs of Figure 3a,b show a difference in the maximum concentration of CO_2_ depending on the ventilation system operation mode, and a difference in the overall rate of decrease in the CO_2_ concentration after the occupants leave. The comparison between the CO_2_ concentration change and the amount of change in the mean value of the CO_2_ concentration for 15 min shows that the CO_2_ concentration change had significant noise, but the amount of change in the mean value of the CO_2_ concentration for 15 min had relatively little noise. Furthermore, the fine noise has been reduced in the graphs showing the CO_2_ concentration and mean value of the CO_2_ concentration for 15 min.

**Figure 5:**
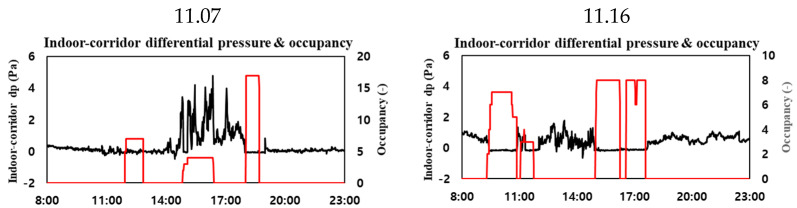
Checking the room–corridor differential pressure and occupancy data on 11/7 and 11/16.

The indoor–corridor differential pressure was equalized when the door was open. Figure 5 presents a graph comparing the indoor–corridor differential pressure and the number of occupants. On 16 November, the door was left open from 9:30 to 11:00 and from 15:00 to 18:00 when the occupants were indoors. As shown in Figure 5, the pressure difference between the room and corridor was equalized by opening the door while the occupants were in the room. Moreover, fluctuations in the differential pressure were confirmed when the door was closed. As the indoor environment and corridor were maintained at an equal pressure in the afternoon of 7 November, the door was considered to have been opened from 18:00 to 19:00 and used, and to have been closed and used in the morning hours. As such, even in the presence of an occupant, the differential pressure is measured in various ways depending on whether the door is open or not. Therefore, the differential pressure data are considered to have caused an error in the occupant judgment classification model. In future, by comparing the differential pressure data and the door sensor data, we can plan to use them for the CO_2_ concentration-based occupant estimation by considering the effect of the indoor CO_2_ concentration depending on whether the doors and windows are open.

**Figure 6:**
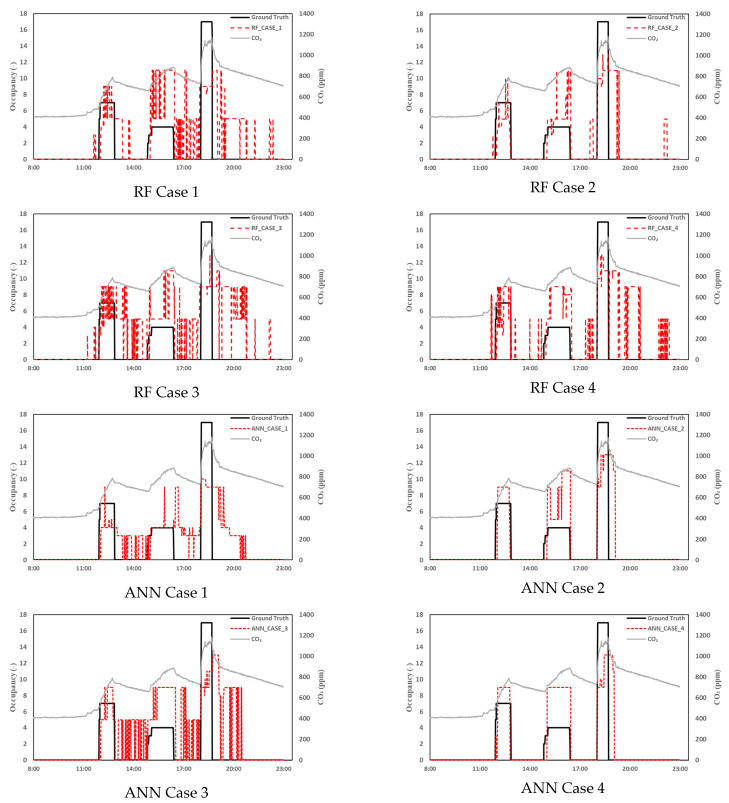
A comparison of the ground truth and estimated occupancy on 11/7.

First, the data between 7 November and 14 November, when the ventilation system was not used, were compared with the actual value and the estimated occupant value output using the RF and ANN. The accuracy and RMSE of the RF are shown in Table 5. Both the Random Forest and ANN models, where the CO_2_ concentration, mean value of the CO_2_ concentration within 15 min, amount of change in the mean value of the CO_2_ concentration within 15 min, and the ventilator operation mode were input, yielded the highest accuracy in Case 2. In the RF, the RMSE error yielded the lowest value at 1.462 in Case 2. In the ANN model, the lowest RMSE value was 1.544 in Case 4, where the CO₂ concentration, mean value of CO_2_ concentration within 15 min, and amount of change in the mean value of the CO_2_ concentration within 15 min were entered. In the model that learned the ventilation operation mode as an input value in a space where the mechanical ventilation was applied, both the RF and ANN models yielded the highest accuracy in Case 2, where the ventilation system operation mode was added as an input value even on days when the ventilation system was not operated (Figure 6). This is because in the remaining cases, except for Case 2 of the RF model and Cases 2 and 4 of the ANN model, when the CO₂ concentration decreased after the occupant had left the room, the learning model determined the presence of an occupant (Figure 6). In Case 2, where the ventilator operation mode was used as an input value, such a problem did not occur and the accuracy was inferred to be relatively high. In Case 2 of the RF model and Cases 2 and 4 of the ANN model, a situation occurred when numerous occupants left simultaneously, but it was determined that some occupants remained. However, this situation did not occur when the number of occupants was small. Case 3, where the indoor–outdoor and indoor–corridor differential pressure data were added as input values, yielded the lowest accuracy and highest error in the RF and ANN models. Figure 6 shows that compared to other case models, the Case 3 model determines the occupants to be present even after they have left. The RMSE of Case 4 in the ANN model is lower than that of Case 2 because Case 2 of the ANN model fits the occupancy range better than Case 4 throughout the study period. However, Case 2 estimates the number of occupants more within the same range. Therefore, the RMSE for Case 2 exceeded that for Case 4. In future studies, to address this issue, we will accumulate data to determine and increase the accuracy using the number of occupants rather than the occupancy range.

**Figure 7:**
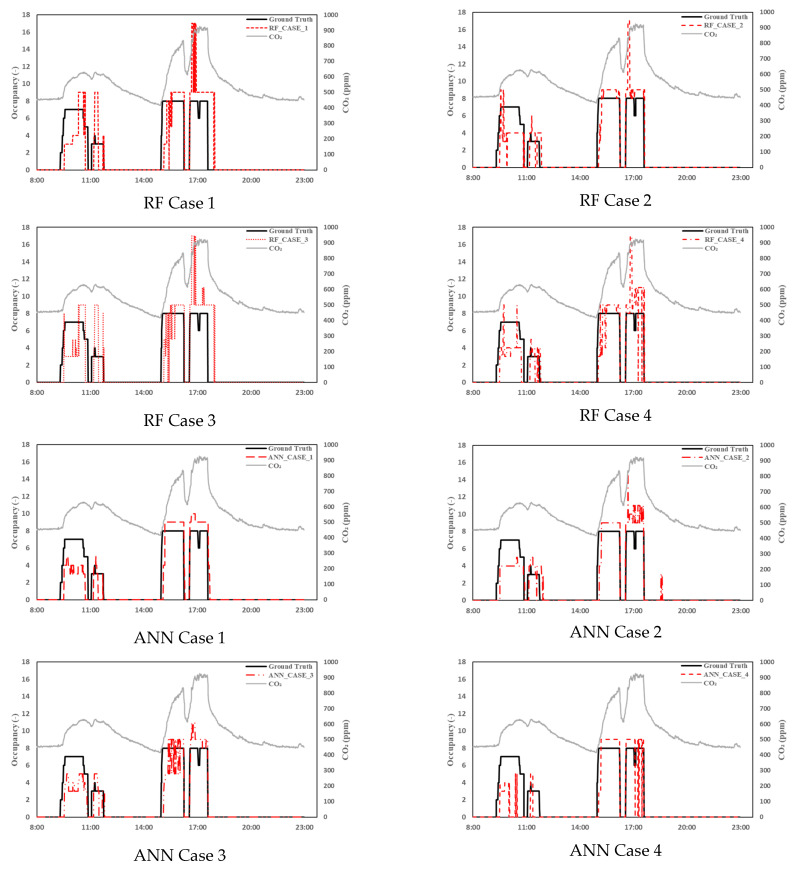
A comparison of the ground truth and estimated occupancy on 11/16.

The second data set covered the entire validation period from 7 November to 22 November. From 15 November to 22 November, the occupants using the room operated the ventilation system arbitrarily. Table 6 shows the results of comparing the RMSE of the Random Forest and ANN model for each case using the data from 7 November to 22 November when the ventilation system was used randomly. In Figure 7, as in Figure 6, the accuracy of the number of occupants in Case 2 of the two models throughout the data range for verification yielded the highest values in both the RF model (0.9102) and the ANN model (0.9180). Figure 7 shows the data for 16 November. On this day, the ventilation system was operated in mode 1 from 09:30 and mode 3 from 14:59. Unlike 7 November, when the ventilation system was not operated, on this day, when the ventilation system was turned on, the CO_2_ concentration rapidly decreased after the occupants left. In all cases, there was no state of determining that there were occupants when the CO_2_ concentration slowly decreased after the occupants left. A comparison of the case of the RF model and that of the ANN model in Figure 7shows that when an occupant re-enters an hour after an occupant leaves and the CO_2_ concentration rises rapidly, the RF model shows that there are more than 10 occupants. Inferences were made in numerous situations, but in the case of the ANN, more occupants were estimated in relatively fewer situations. Comparing the accuracy of the RF and the ANN models, in all cases, the accuracy of the ANN model was relatively high. The RMSE was relatively low in the ANN except for Case 2. In addition, the RMSE yielded the smallest error 1.743 in Case 2 only when the ventilation system was running.

Excluding the occupants, factors that affect the indoor CO_2_ concentration are the ventilation system operation mode, infiltration amount, and differential pressure. The accuracy could be improved when estimating the number of occupants, but in estimating the number of occupants based on the CO_2_ concentration, the pressure difference between the room and the surrounding space and the adjacent CO_2_ concentration did not contribute to increasing the accuracy of the model. Based on the case results of the Random Forest and ANN models, the accuracy of the occupant estimation model based on the CO_2_ concentration can be improved by adding the ventilation system operation mode as an input value.

In a room where a mechanical ventilation system is installed and the ventilation system operation and recirculation air ratio are adjusted to reduce energy consumption, the ventilation system operation mode has a significant effect on the change in CO_2_ concentration. Based on the above results, when estimating the number of occupants based on the CO_2_ concentration in a room where a ventilation system is installed, adding the ventilation system operation mode as an input value can improve the estimation accuracy.

## 5. Discussion

Studies [12,13,14,15,16,17,18] on the estimation of the number of occupants based on CO_2_ have used data from various sensors such as Wi-Fi probes, sound, and PIR sensors along with the CO_2_ concentration. The Wi-Fi probe can generate issues related to personal information. The PIR data are limited by the possible reduction in accuracy owing to errors such as data accumulation. Sound data can occur instantaneously or be greatly influenced by the surroundings. In this study, to estimate occupancy and manage indoor air quality environmental factors such as the indoor–outdoor and indoor–corridor differential pressure, the ventilation system operation was added to present a method for estimating the number of occupants using a CO_2_-based machine learning model. Estimating the number of occupants can be beneficial for indoor air quality management and infection control; in particular, this estimation can help to determine the frequency of ventilation for a space used by several people or the level of mechanical ventilation that should be performed when the utilization rate is highest. This helps determine the ventilation method that should be adopted when there is an unspecified number of users or an infection is suspected among the occupants. Currently, a method for estimating the occupancy of a single room is being studied; the measurement and application of data in various large scale spaces can allow for the efficient management of large commercial buildings.

However, the model presented in this study was based on one target room and the amount of learning data is considered to be insufficient as an initial model. It is possible to estimate the opening of the doors and windows using the pressure differential, but there is no accurate data on the open state of the doors and windows, and they can be opened freely according to the judgment of the occupant. By establishing that the differential pressure data and ventilation system operation can influence the accuracy of the occupancy estimation, the acquisition of long-term data is improved and the possibility of model development is enhanced. As mentioned earlier, applying the model to large spaces would facilitate an improved efficiency in building management (energy, indoor air quality, and infection control, etc.). The accuracy should be improved by considering the additional data and various variables, and because the differential pressure is affected by the space–building entry/exit conditions (wind speed, wind direction, weather, and season, etc.), it should further be improved through seasonal data acquisition and learning.

As the differential pressure between the indoor and the adjacent space increases, the amount of air leakage also increases. Accordingly, this study tried to accurately determine the change in the indoor CO_2_ concentration according to the occupancy, by using the differential pressure between rooms and adjacent space. However, when the door is open, the differential pressure becomes an equal pressure, but the air exchange rate between the corridor and the indoor space increases compared to when the door is closed. The differential pressure data should be included depending on whether the door is open or not. In addition, the positive pressure and negative pressure conditions should be considered separately. Under a positive pressure, where the pressure in the room is higher than the adjacent pressure, the CO_2_ concentration in the adjacent room does not affect the CO_2_ concentration in the room because the airflow is formed from the room to the adjacent one. Under a negative pressure, where the pressure in the room is lower than the adjacent pressure, the airflow is formed from the adjacent space to the room, and the adjacent CO_2_ concentration affects the indoor CO_2_ concentration. The CO_2_ concentration in the room is estimated by dividing it according to the value of the indoor and adjacent differential pressure. For the same reasons as above, adding the differential pressure as an input value in this paper is considered to reduce the accuracy.

For future applications in various spaces, the air change per rate will be considered. To include the size of the space, the number of occupants can be applied in various spaces if learning is conducted using the occupied space per person or the number of occupants per unit space. The differential pressure data is intended to be applied to machine learning according to the positive and negative pressure values.

## 6. Conclusions

In this study, CO_2_-based machine learning using differential pressure data and reflecting the influence of the ventilation system operation mode was used to estimate the number of occupants in a room. The following conclusions were drawn from the study findings:

(1) The ventilation system operation data increased the estimation accuracy in the RF and ANN models. In the RF model, the RMSE increased from a maximum of 0.898 to 0.9198 depending on the ventilation system operation. The RMSE was lowest in the RF model where the ventilation system operation data were added as input values;
(2) The addition of the differential pressure data as input data decreased the accuracy and increased the RMSEs in the RF and ANN models. The differential pressure data were considered to have an effect on the CO_2_ concentration, but were not considered to be related to prediction of the occupancy;
(3) The accuracy of estimating the number of occupants based on the CO_2_ concentration using machine learning can be improved by adding the ventilation system operation mode as input data if the mechanical facility operates indoors.

Future studies will regard the air change rate indoors as an input value and also measure environmental and differential pressure data in various spaces using mechanical ventilation. These data will be modified for application in various spaces, not only being applicable in models for specific spaces. The aim is to improve the accuracy of the model by continuously measuring data in the living-lab, thus increasing the amount of data. In addition, accuracy is compared using other machine learning models and learning models are optimized. For the differential pressure data to affect the increased accuracy of occupant estimation, the amount of change in the mean value of CO_2_ concentration for 15 min, and the CO_2_ concentration of the surrounding space and the differential pressure data need to be related.

## Figures and Tables

**Table 1:** Previous research on the CO_2_ concentration based on the occupancy estimation.

	Input Data	ML Model	Accuracy Factors
[5]	CO₂, temperature, humidity, pressure	ELM	Accuracy
[6]	CO₂, temperature, humidity, pressure	ELM, SVM, ANN, LDA, kNN, CART	Accuracy NRMSE
[7]	CO₂, temperature, humidity	GB, kNN, LDA, RF	Accuracy RMSE NRMSE CV
[8]	CO₂	ELM	RMSE
[9]	CO₂, 15 min CO₂ concentration mean, 15 min CO₂ concentration mean variation, indoor-outdoor CO₂ concentration ratio	CART, HMM	RMSE Accuracy
[10]	CO₂, CO₂ variation, 5 min CO₂ concentration mean	SVM, ANN, PEM	RMSE Fb
[11]	CO₂	GCForest, SVM, CART, IHMM	EA MAE DA
[12]	CO₂, temperature, humidity, light,	LDA, CART, RF, GBM	Accuracy
[13]	CO₂, temperature, light, PIR, sound	LDA, QDA, SVM, RF	Accuracy f1 score
[14]	CO₂, temperature, humidity, pressure, TVOC, sound, window state	FNN	Accuracy
[15]	CO₂, light energy consumption, temperature, humidity	LDA, RF, CART	Accuracy
[16]	CO₂, temperature, humidity, Wi-Fi probe	kNN, SVM, ANN	MAE RMSE MAPE
[17]	CO₂, Wi-Fi probe, PIR, energy consumption	ANN	R^2^ NRMSE
[18]	environmental sensing, camera, Wi-Fi probe	ANN	MAE f1 score
[19]	-	LSTM, RNN	MAPE RMSE MAE
[20]	energy consumption	RF, SVM, kNN, ANN, GB	Accuracy Precision
[21]	CO₂	GCForest, HMM	EA DA
[22]	CO₂, temperature, humidity, light	Navie Bayes Classification via Regression Decision Table RF Simple Logistic Multi Class Classifier	-
[23]	Indoor and outdoor environmental sensing, Wi-Fi probe, Energy consumption	DNN, LSTM, Bi-LSTM, GRU, Bi-GRU	-
[24]	CO₂, light sensing	-	-
[25]	CO₂, trend value, seasonal value, Irregular value	-	Accuracy
[26]	CO₂, temperature, humidity, dew point	SVM, AdB, RF, GB, LR, MLP	RMSE MAE MAPE R^2^
[27]	CO₂, temperature, humidity, air pressure	CDBLSTM	Accuracy NRMSE

**Table 2:** The Internet of Things sensor specification.

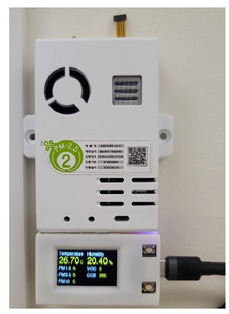	CO₂	Model: CM1106-C CO₂ 0~5,000 ppm ± (50 ppm + 5% of reading)	CUBIC China
	PM	Model: PM2008 PM 0~1000 μg/m^3^ 0~100 ug/m^3^, ±1 0 ug/m^3^ 101~1000 ug/m^3^, ± 10% reading	CUBIC China
	Temperature	Model: Si7021-A20 Temperature −40 °C~85 °C ± 0.3 °C	SILICON LABS USA
	Relative Humidity	Model: Si7021-A20 Relative Humidity 5~95% ± 2% RH	SILICON LABS USA
	VOCs	Model: SP3S-AQ2-01	NISSHA Japan
	MCU	Model: TTGO-T-Display ESP32	LILYGO China
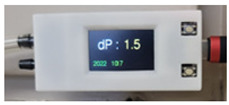	Differential pressure	Model: D6F-PH0505AD3 DP ± 50 Pa	OMRON Jaan
	MCU	Model: TTGO-T-Display ESP32	LILYGO China

**Table 3:** The cases of machine learning.

	CO_2_ Concentration (ppm)	Mean Value of the CO_2_ Concentration for 15 min (ppm)	Amount of Change in the Mean Value of the CO_2_ Concentration for 15 min (ppm)	Differential Pressure Indoor and Corridor (Pa)	Differential Pressure Indoor and Outdoor (Pa)	Corridor CO₂ Concentration(ppm)	Ventilation System State (-)
Case1	O	O	O	O	O	O	O
Case2	O	O	O	X	X	X	O
Case3	O	O	O	O	O	O	X
Case4	O	O	O	X	X	X	X

**Table 4:** Results of the random forest and artificial neural network self-test.

	RF Accuracy	RF RMSE	ANN Accuracy	ANN RMSE
Case1	0.9757	0.8178	0.9745	1.177
Case2	0.9635	0.9018	0.9674	1.184
Case3	0.9696	0.8705	0.9647	1.219
Case4	0.9502	0.9940	0.9720	1.332

**Table 5:** The Accuracy and RMSE results of all cases from 11/7 to 11/14.

	RF-Accuracy	RF-RMSE	ANN-Accuracy	ANN-RMSE
Case1	0.9182	1.925	0.9275	1.775
Case2	0.9198	1.462	0.9283	1.618
Case3	0.8951	2.353	0.8898	2.072
Case4	0.8980	1.840	0.9130	1.544

**Table 6:** The Accuracy and RMSE results of all cases from 11/7 to 11/22.

	RF-Accuracy	RF-RMSE	ANN-Accuracy	ANN-RMSE
Case1	0.9061	1.915	0.9155	1.750
Case2	0.9102	1.743	0.9180	1.770
Case3	0.8916	2.134	0.8936	2.042
Case4	0.9003	1.933	0.9147	1.787

## Data Availability

Not applicable.
